# Destiny of Spermatozoa in Continent Adults

**Published:** 1912-10

**Authors:** 


Destiny of Spermatozoa in Continent Adults. Leuret and Eskuier, ibid., Jan 28, 1912, deny that spermatorrhœa occurs except after sexual "approach" or a distinct emission. They find in the urine polychromatophile particles which they regard as debris of the heads of the spermatozoa. Brandeis questions whether these are not the initial stage in the formation of spermatozoa, the normal process of maturiation not continuing.

